# PlexinA1 activation induced by β2-AR promotes epithelial-mesenchymal transition through JAK-STAT3 signaling in human gastric cancer cells

**DOI:** 10.7150/jca.70000

**Published:** 2022-04-18

**Authors:** Ying Liu, Yanhui Hao, Hanzheng Zhao, Ying Zhang, Die Cheng, Li Zhao, Yuqiao Peng, Yanjie Lu, Yuhong Li

**Affiliations:** 1 Department of Pathology, Chengde Medical University, Chengde, Hebei, China.; 2 Cancer Research Laboratory, Chengde Medical College, Chengde, Hebei, China.; 3 Beijing Institute of Radiation Medicine, Beijing, China.; 4 Department of General Surgery, The Second Affiliated Hospital of Harbin Medical University, Harbin, Heilongjiang, China.; 5 Department of clinical laboratory, The First Affiliated Hospital of Chengde Medical College, Chengde, Hebei, China.; 6 Department of Ultrasound Medicine, The First Affiliated Hospital of Chengde Medical College, Chengde, Hebei, China.

**Keywords:** gastric cancer, chronic stress, epithelial-mesenchymal transition, β2-AR, PlexinA1, JAK-STAT3

## Abstract

With the medical model shifting from a single biomedical model to a biopsychological-social model, the impact of psychosocial factors on cancer patients has attracted attention. Studies have shown that chronic stress caused by long-term psychological stress, such as anxiety and depression, can promote the malignant progression of tumors by acting on β2-adrenergic receptor (β2-AR). β2-AR can promote tumor migration by activating epithelial-mesenchymal transition (EMT). However, the underlying mechanisms in the regulation of EMT by β2-AR are still unclear. In this study, we established a chronic stress model by treating MGC-803 and SGC-7901 human gastric cancer cells with isoproterenol (ISO), a β2-AR agonist. EMT in the two gastric cancer cell lines was enhanced after ISO treatment. Thereafter, we found that the interaction between β2-AR and PlexinA1 was involved in the process by which chronic stress affects EMT in both MGC-803 and SGC-7901 cells. Moreover, the activation of β2-AR by ISO increased the expression of PlexinA1, activated JAK-STAT3 signaling and further promoted EMT in human gastric cancer cells. Importantly, the knockdown of PlexinA1 by small hairpin RNAs inhibited JAK-STAT3 signaling and abolished the EMT induced by β2-AR. In conclusion, PlexinA1 was an important downstream target of β2-AR, through which β2-AR promoted EMT in human gastric cancer cells by activating JAK-STAT3 signaling.

## Introduction

Gastric cancer has become a major disease that seriously endangers human health. According to the latest statistics, there will be more than 1 million new cases of gastric cancer in the world, and the number of deaths will reach 760,000 in 2020, accounting for the fifth and fourth highest number of diagnosed cancer cases and deaths worldwide 1. Chronic stress is a recognized risk factor that promotes the occurrence and development of tumors through the release of stress hormones (such as adrenaline and norepinephrine, etc.) 2. Many studies have shown that the activation of β2-AR is an important mechanism by which chronic stress affects tumor progression. EMT plays an important role in the initial stage of tumor invasion and metastasis, and β2-AR can promote tumor migration by activating EMT 3-5. However, the underlying mechanisms by which β2-AR regulates EMT are still unknown.

As members of the Sema family, plexins can be divided into four subtypes, Plexin A (1-4), Plexin B (1-3), Plexin C1 and Plexin D1, and were first discovered to play critical roles in nerve axon guidance 6. PlexinA1 works independently by binding to corresponding ligands 7. Our previous study found that the activation of β2-AR induced by ISO could significantly increase the expression of PlexinA1 in gastric cancer cells 8. Therefore, we speculated that PlexinA1 might be an important downstream molecular target of β2-AR and be involved in the malignant progression of tumors induced by chronic stress. However, whether and how PlexinA1 regulates tumor progression affected by chronic stress has not yet been studied. The JAK-STAT (Janus kinase-signal transducer and activator of transcription) signaling pathway plays a key role in the EMT process of tumor cells 9, 10. In the process of chronic stress, whether β2-AR/PlexinA1 regulates EMT in gastric cancer cells through the JAK-STAT3 signaling pathway needs to be further explored.

In this study, we first established a chronic stress model by treating MGC-803 and SGC-7901 human gastric cancer cells with ISO. Then, we confirmed that PlexinA1 interacted with β2-AR by immunofluorescence and coimmunoprecipitation. On that basis, we observed the regulatory role of β2-AR on PlexinA1, EMT and the JAK-STAT3 signaling pathway through β2-AR functional enhancement and inhibition by chemical methods. Finally, we knocked down PlexinA1 by applying small interfering RNAs and studied its role in β2-AR-JAK-STAT3-mediated EMT in gastric cancer cells.

## Materials and methods

### *In vivo* tumorigenicity assay

A total of 15 male BALB/c nude mice (6-8 week-old, body weight 18-20g) were provided by Huafukang Bioscience Co., Ltd. (Beijing, China) and housed within the individually ventilated caging systems. The mice were given subcutaneous injection of MGC803-luc gastric cancer cell suspension (200 μl; 1×10^6^ cells/ml) in the posterior region, and the tumor size was measured by IVIS lumina II (Perkin Elmer) imaging system 28 days later. Luciferase activity was measured after intraperitoneal injection of 10 ul/g D-luciferin (15 mg/ml). After that, the mice were sacrificed for further tests.

### Cell culture and drug treatment

Human gastric cancer cell lines MGC-803 was brought from the Shanghai Genechem Co., Ltd. (GENE, Shanghai, China) and SGC-7901 was purchased from the Shanghai Biowing Applied Biotechnology Co., Ltd. (BIOWING, Shanghai, China). The cells were cultured in Dulbecco's modified Eagle's medium (DMEM, Gibco, Grand Island, NY) supplemented with 10% fetal bovine serum (FBS, Gibco, Grand Island, NY). When the cell density reached 70%, the cells were treated with 2 μM, 10 μM, or 20 μM ISO (HARVEST, Shanghai, China) or 20 μM, 40 μM, or 50 μM ICI118,551 (a specific β2-AR antagonist) (Abcam, #ab120808, US), while the control group was given matching normal saline. MGC-803 cells and SGC-7901 cells were harvested and prepared for the next trial after 12 hours and 24 hours, respectively.

### ELISA Assay

Blood samples from BALB/c nude mice were collected for 15 min of centrifugation at 3000 ×g under 4 °C. Mouse serum adrenocorticotropic hormone (ACTH) and epinephrine levels were tested using the corresponding mouse ELISA kit (Mlbio biotech company, Shanghai, China) in accordance with specific protocols. In short, the microplate reader was used to measure absorbance 450 nm.

### Western blotting

MGC-803 and SGC-7901 cells were lysed in a radio immunoprecipitation assay (RIPA, Solarbio, Beijing, China), and total proteins were extracted. The protein concentration was measured using a BCA Protein Assay kit (Thermo Scientific, #23,227, USA). Protein expression was detected by rabbit anti-β2-AR antibody (1:5,000 dilution, Abcam, #ab182136,US), anti-PlexinA1 (1:500 dilution, HUABIO, #ET1706-17, Hangzhou, China), anti-JAK2 (1:1000 dilution, HUABIO, #ET1607-35, Hangzhou, China), anti-P-JAK2 (1:500 dilution, HUABIO, #ET1607-34, Hangzhou, China), anti-STAT3 (1:1000 dilution, HUABIO, #ET1607-38, Hangzhou, China), anti-p-STAT3 (1:1000 dilution, HUABIO, #ET1603-40, Hangzhou, China), anti-E-cadherin (1:500 dilution, HUABIO, #0407-25, Hangzhou, China), anti-N-cadherin (1:5000 dilution, HUABIO, #ET1607-37, Hangzhou, China), anti-α-SMA (1:5000 dilution, HUABIO, #ET1607-43, Hangzhou, China), anti-Snail (1:1000 dilution, HUABIO, #ER1706-22, Hangzhou, China), anti-ZEB-1 (1:2000 dilution, ABclonal, #A5600, Wuhan, China), anti-Slug (1:2000 dilution, ABclonal, #A1057, Wuhan, China), anti-Vimentin (1:5000 dilution, HUABIO, #ET1610-39, Hangzhou, China), rabbit antibody, anti-β2-AR (1:1000 dilution, Santa Cruz Biotechnology (CBST), sc-271822, US) and anti-glyceraldehyde-3-phosphate dehydrogenase (GAPDH, 1:10,000 dilution, Abcam, #ab8245, US) mouse antibody, at 4 °C overnight. The membranes were washed and incubated with corresponding goat anti-rabbit IgG-horseradish peroxidase (HRP) (1:10,000 dilution, ZSGB-BIO, #ZB-2301, Beijing, China) and goat anti-mouse IgG-HRP (1:10,000 dilution, ZSGB-BIO, #ZB-2305, Beijing, China) at room temperature for 1 hour. Finally, the membrane was treated with enhanced chemiluminescence (ECL, Vazyme, #E-411-05-AB, Nanjing, China) and imaged using a chemiluminescence imaging system (Clinx Science Instruments, ChemiScope 6100, Shanghai, China). ImageJ software (ImageJ 1.53c, National Institutes of Health, US) was used to calculate the gray value of the protein bands, and the protein expression was normalized to GAPDH.

### Immunofluorescence staining

MGC-803 cells and SGC-7901 cells were grown on coverslips. At the appropriate time points, the cells were harvested and fixed with 4% paraformaldehyde. The cells were labeled with rabbit anti-PlexinA1 (1:200 dilution, HUABIO, #ET1706-17, Hangzhou, China), mouse anti-β2-AR (1:200 dilution, Santa Cruz Biotechnology (CBST), sc-271822, US), rabbit anti-E-cadherin (1:500 dilution, GeneTex, #GTX100443, Southern California, US), rabbit anti-α-SMA (1:200 dilution, HUABIO, #ET1607-43, Hangzhou, China), and rabbit anti-Vimentin (1:200dilution, HUABIO, #ET1610-39, Hangzhou, China) overnight at 4 °C. After that, the samples were incubated with the corresponding fluorescein isothiocyanate (FITC)-labeled goat anti-mouse secondary antibody (1:2000 dilution, ab150113, Abcam, US) and tetramethylrhodamine isothiocyanate (TRITC)-labeled goat anti-rabbit secondary antibody (ab150080, Abcam, US). 4',6-Diamidino-2'-phenylindole (DAPI, Vectorlabs, H-1200-10, San Francisco, US) was used to stain the nuclei. Cells were subsequently scanned with a fluorescence microscope (UltraVIEW VOX, PerkinElmer, US).

### Transwell migration analysis

Transwell chambers (6.5 mm diameter inserts; 8 μm pore size; polycarbonate membrane, CORING, USA) were used to detect cell migration. The cells were collected and resuspended in serum-free DMEM at a concentration of 0.5×10^5^ cells/ml. Then, the cell suspension solution was seeded into the upper chambers (200 μl per well). The bottom chambers were filled with 1 ml of cell culture medium (serum free) containing 20 μM ISO or 50μM ICI. After being cultured at 37 °C for 24 h, the cells that did not penetrate the polycarbonate membrane were wiped off with cotton buds. The membrane was removed and fixed with 4% paraformaldehyde and stained with crystal violet solution. Five fields were randomly selected under an Olympus microscope (Tokyo, Japan), and the number of cells was counted.

### Scratch test

MGC-803 cells and SGC-7901 cells were cultured in six-well plates at 37 °C. When the cell density reached 80%, scratch wounds were made using the fine end of 10 μL pipette tips in the middle. Images of the migrated cells at 0 h and 24 h were captured under a phase-contrast microscope. Three independent repeated tests were conducted.

### PlexinA1 knockdown with shRNA

MGC-803 cells and SGC-7901 cells were incubated with LV3 (H1/GFP&Puro)-shPlexinA1 (GCAGCGAGCAGTTTGTCTACT, GenePharma, Shanghai, China) and LV3 (H1/GFP&Puro)/Neo-shNC (GCACACCCATCGATGGCAAGT, GenePharma, Shanghai, China) for 48 hours. The experiment was performed according to the manufacturer's protocols. The infection rate of the lentivirus was analyzed by flow cytometry. Then, 20 μM ISO or matching normal saline was added. After 12 hours, the cells were harvested for the next trial.

### Coimmunoprecipitation (Co-IP) assay

MGC-803 cells and SGC-7901 cells were washed twice with 4 °C phosphate-buffered saline (PBS, Gibco, Grand Island, NY), lysed with IP lysis buffer (Minute™ Non-Denatured Protein Solubilization Reagent Cat. No. WA-010) for 30 min, and centrifuged at 12,000 rpm/min for 10 min. The supernatant was collected. Protein A + G-agarose was washed three times with washing buffer, and extracts were incubated for 2 h at 4 °C with IgG and protein A + G-agarose to eliminate nonspecific binding. Primary antibodies against PlexinA1 and β2-AR were then added to separate cell extract tubes for incubation at 4 °C overnight. Protein A/G was added and incubated with rotation for 8 h at 4 °C. Protein A/G-agarose was collected by centrifugation. The supernatant was discarded after washing and resuspended in 50 μl of 1× loading buffer, and the supernatant was moved to a new tube and boiled in a 100 °C metal bath for 10 min. Immunoprecipitated proteins were analyzed by Sodium dodecyl sulfate polyacrylamide gel electropheresis (SDS/PAGE) and immunoblotted with anti-PlexinA1 (Rb, 1:500), anti-β2-AR (Ms, 1:1000), anti-GAPDH (Ms, 1:10000) and corresponding secondary antibodies. The protein bands were detected using a chemiluminescence imaging system.

### Statistical analysis

All tests were repeated at least 3 times. The data are presented as the mean±s.d. The statistical analyses were conducted using GraphPad Prism version 5 (GraphPad software, San Diego, CA). All data from different groups were compared after one-way ANOVA. Differences were considered significant at the level of a two-sided P < 0.05.

## Results

### *In vivo* analysis of ISO and ICI118,551 in regulating tumor growth of gastric cancer cells

**Figure 1:**
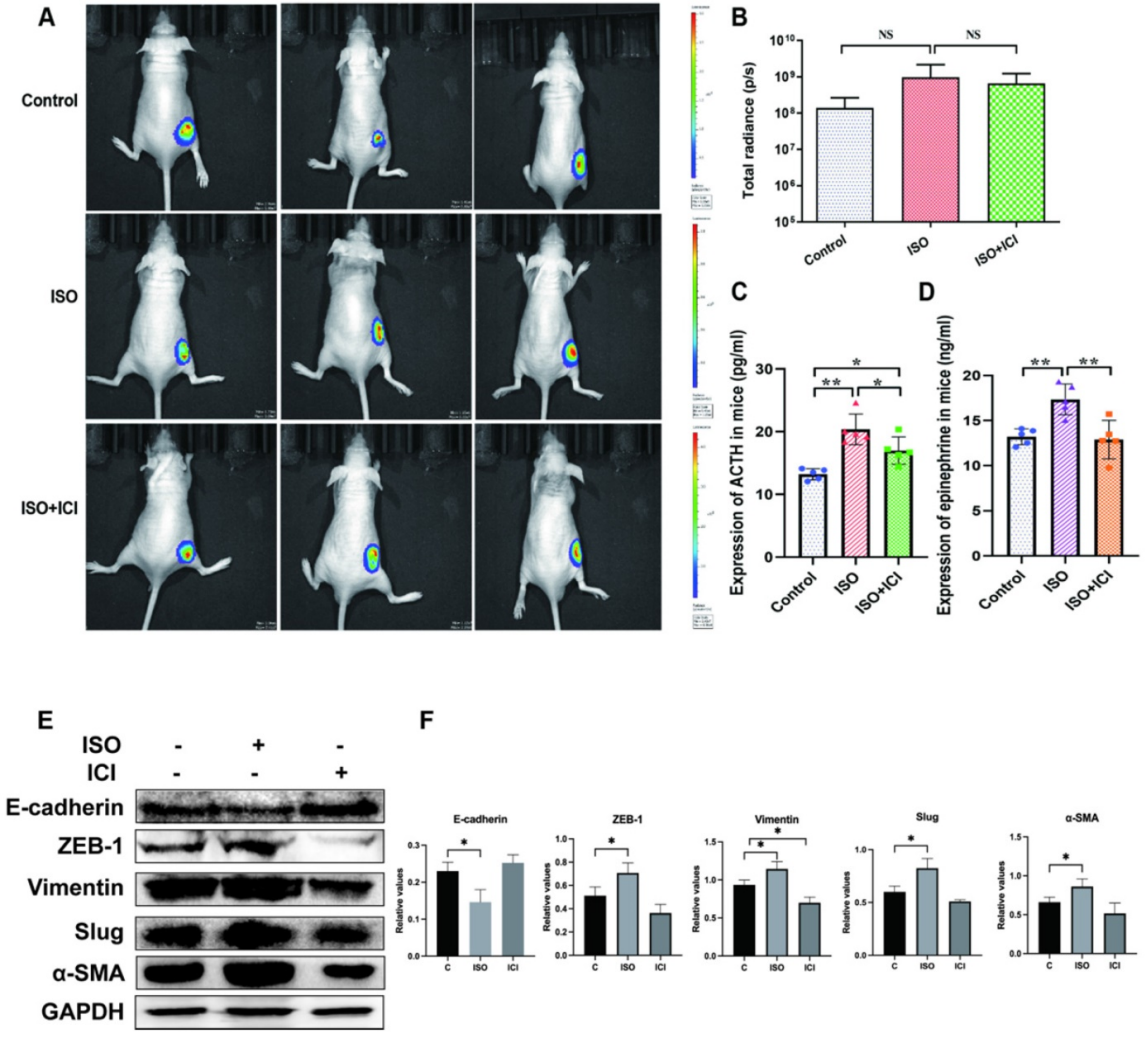
** Mice were treated with ISO (5 mg/kg/d) and ICI118,551 (0.2 mg/kg/d). (A)** Representative images of bioluminescence from mice with MGC803-Luc tumors in 3 groups. **(B)** Quantification of bioluminescence from mice with MGC803-Luc tumors. **(C)** Mouse serum ACTH levels. **(D)** Mouse serum epinephrine levels. **(E-F)** Mouse were treated with ISO and ICI118,551 and the tumor were collected for examination. The protein levels of E-cadherin, ZEB-1, Vimentin, Slug and α-SMA were tested by western blotting. Three independent repeated experiments were conducted. The gray values of the protein bands were analyzed through ImageJ software, relative values to GAPDH were calculated. Data represent mean ± Standard Deviation. (s.d) (n=6 for each group), *P<0.05, **P<0.01.

We first validated whether ISO affects tumor growth *in vivo*. However, *in vivo* imaging analysis showed that ISO did not affect the ability of tumor growth, and ICI118,551 did not inhibit the tumor growth in nude mice (Fig. 1A-B). This finding consistent with our previous study 11 and another study by Caroline P. Le1 12, which suggested no influence of chronic stress on the growth of primary tumor. Interestingly, we found that ACTH level in ISO group was significantly higher than that in the control group, whereas ICI118,551 treatment reversed this increase (Fig. 1C). It suggests that the Hypothalamic- Pituitary- Adrenal axis (HPA axis) remains active in ISO treatment. In this study, we further tested the change of serum epinephrine levels. The epinephrine levels in ISO group were significantly increased compared with those in the control group, whereas ICI118,551 treatment reversed this increase (Fig. 1D). Thereafter we detected the expression of EMT markers in mouse tumor tissues. We found that the expression of epithelial marker E-cadherin was downregulated, while the expression of mesenchymal markers, including ZEB-1, Vimentin, Slug and α-SMA, were upregulated, indicating the enhanced EMT in tumor issuses pretreated with ISO (Fig. 1E and F). These results suggest that ISO treatment appears to affect the progression of gastric tumors through the regulation of EMT, but not tumor growth.

### β2-AR positively regulates EMT in human gastric cancer cells

**Figure 2:**
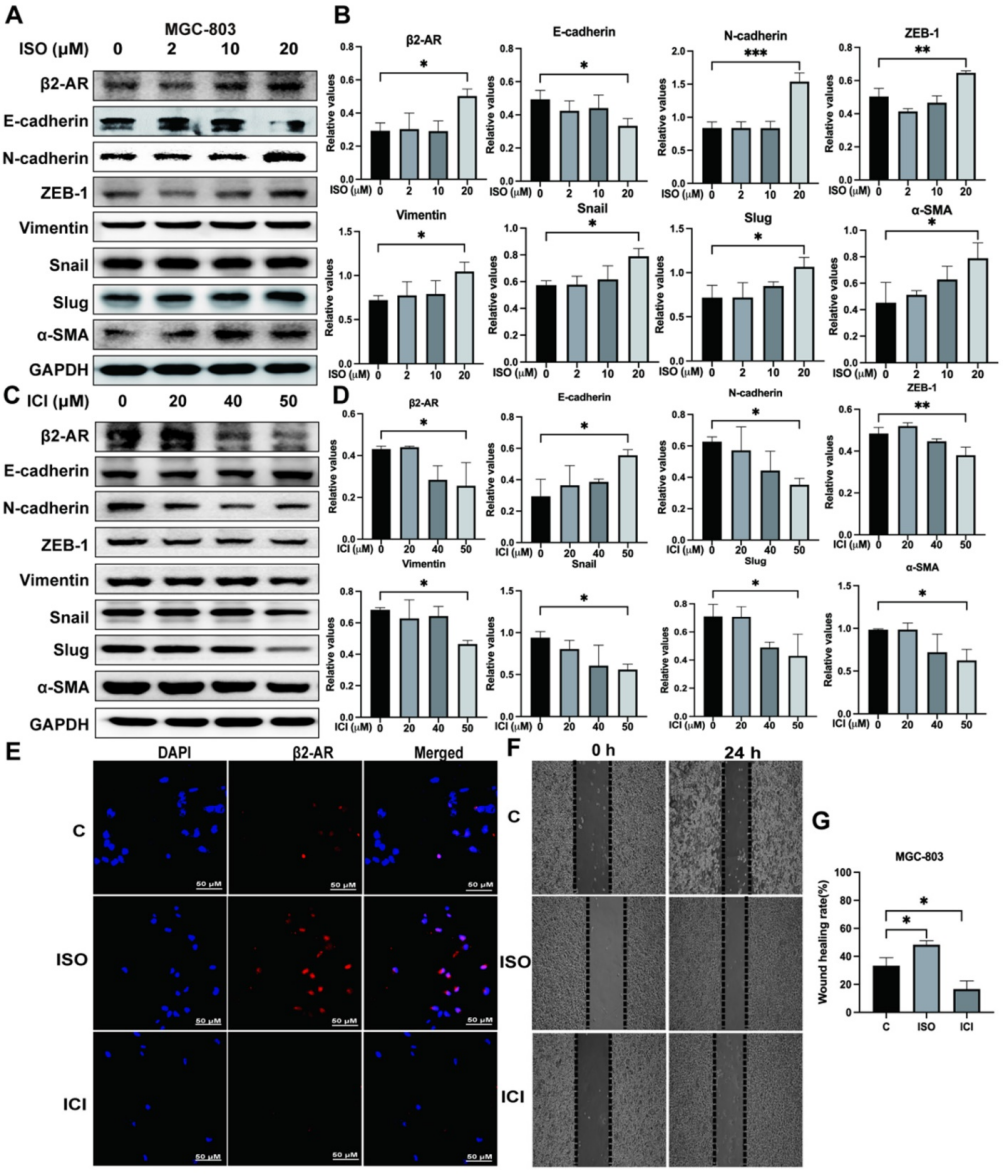
** Effects of isoproterenol (ISO) and ICI118,551 (ICI) on the expression of β2-AR, epithelial markers (E-cadherin) and mesenchymal markers (N-cadherin, ZEB-1, Vimentin, Snail, Slug and α-SMA). MGC-803 cells were treated with 0, 2 µM, 10 µM, or 20 µM ISO or 20 µM, 40 µM, or 50 µM ICI, and the cells were collected for detection 12 hours later.** The protein levels of β2-AR, E-cadherin, N-cadherin, ZEB-1, Vimentin, Snail, Slug and α-SMA were detected by western blotting. Three independent repeated experiments were conducted. The gray values of the protein bands were analyzed through ImageJ software, relative values to glyceraldehyde-3-phosphate dehydrogenase (GAPDH) were calculated. The protein bands and associated statistical analysis data in the ISO group are shown in **(A-B)**, and those in the ICI group are presented in **(C-D)**. In addition, the expression of β2-AR in MGC-803 cells treated with ISO and ICI was confirmed by immunofluorescence, and representative images are shown in **(E)**. Finally, a scratch test was performed to study the migration ability of MGC-803 cells. Scratch wounds were made by using the fine end of 10 µL pipette tips, and images were acquired immediately and 24 hours later by a light microscope. Representative images are shown in **(F).** The width of the scratch was measured, and the mobility rate [mobility rate = (width at 0 h -width at 24 h)/width at 0 h] was calculated. The statistical analysis of 3 repeats is shown in **(G)**. The data are presented as the mean±s.d. *, P < 0.05; **, P < 0.01; ***, P < 0.001.

Studies have shown that β2-AR is an important effector molecule for chronic stress-mediated adverse effects on tumor progression 8, 13, 14. EMT is an important factor affecting tumor invasion and metastasis 15-18. In our study, we treated MGC-803 cells with ISO, a nonselective β-adrenergic receptor (βAR) agonist of β2-AR, at different concentrations and found that 20 μM ISO obviously upregulated the expression of β2-AR (Fig. 2A and B). Moreover, the expression of E-cadherin, an epithelial marker, was downregulated, while the expression of mesenchymal markers, including N-cadherin, ZEB-1, Vimentin, Snail, Slug and α-SMA, was upregulated, indicating enhanced EMT in MGC-803 cells pretreated with ISO (Fig. 2A and B). On the other hand, when the function of β2-AR was specifically inhibited by ICI118,551, EMT in MGC-803 cells was blocked, as shown by increased epithelial markers and reduced mesenchymal markers (Fig. 2C and D). We also applied immunofluorescence to verify the effects of ISO and ICI on β2-AR expression in MGC-803 cells and found results consistent with those of western blotting (Fig. 2E). After that, we examined the impact of ISO and ICI on MGC-803 cell migration through the cell scratch test. The results showed that ISO promoted the migration of MGC-803 cells, while ICI inhibited migration (Fig. 2F and G). In addition, we performed the same experiments using another human gastric cancer cell line, SGC-7901, and obtained consistent results (Fig. S1). These results provided evidence that the activation of β2-AR could promote the occurrence of EMT, which led to increased migration ability in both MGC-803 cells and SGC-7901 cells.

### PlexinA1 and β2-AR colocalize in space and interact with each other

**Figure 3:**
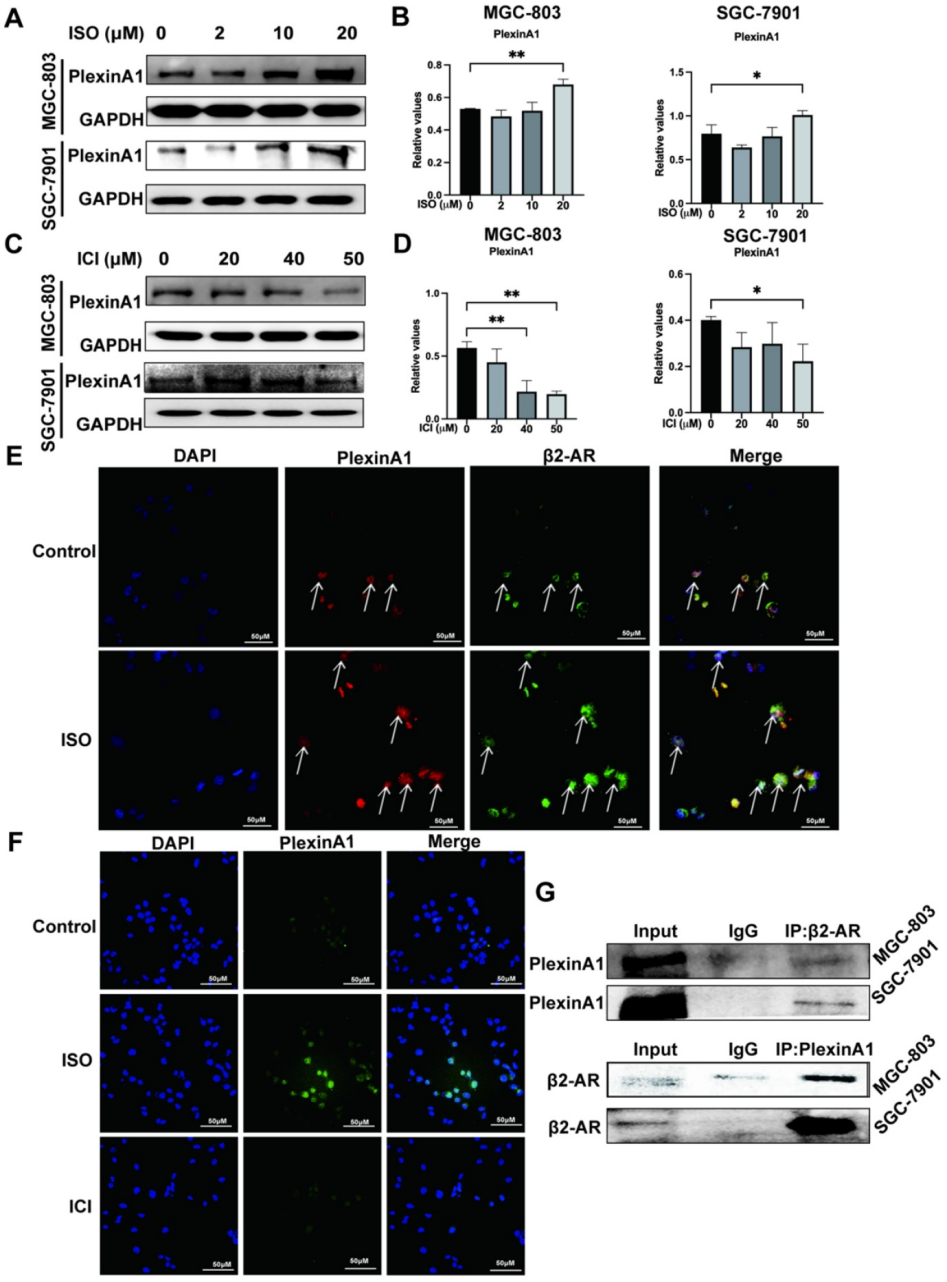
** Impacts of isoproterenol (ISO) and ICI118,551 (ICI) on the expression of PlexinA1. MGC-803 cells and SGC-7901 cells were treated with 0, 2 µM, 10 µM, or 20 µM ISO or 20 µM, 40 µM, or 50 µM ICI for 12 or 24 hours, and the cells were collected for examination.** The protein level of PlexinA1 was tested by western blotting. Three independent repeated experiments were conducted. The gray values of the protein bands were analyzed using ImageJ software, the values relative to glyceraldehyde-3-phosphate dehydrogenase (GAPDH) were computed. The protein bands and associated statistical analysis data in the ISO group and in the ICI group are shown in **(A-D)**. In addition, the expression of PlexinA1 in MGC-803 cells treated with ISO and ICI was verified by immunofluorescence, and typical images are shown in **(F)**. Then, the influence of colocalization between β2-AR and PlexinA1 in MGC-803 cells through immunofluorescence is shown in **(E)**. Finally, coimmunoprecipitation (Co-IP) experiments explored the interaction of β2-AR and PlexinA1 in MGC-803 cells and SGC-7901 cells and are shown in **(G).** The data are presented as the mean±s.d. *, P < 0.05; **, P < 0.01.

We previously found that the expression of PlexinA1 was significantly elevated in the chronic stress model induced by ISO. In the present study, we further explored the regulatory effects of β2-AR on PlexinA1. The results of western blotting and immunofluorescence showed that ISO could activate the expression of PlexinA1 and that ICI could inhibit the expression of PlexinA1 in MGC-803 cells, both of which appeared to be dose-dependent, indicating the important regulatory roles of β2-AR on PlexinA1 (Fig. 3A-D, and Fig. 3F). Moreover, we were surprised to find colocalization between β2-AR and PlexinA1 in MGC-803 cells through immunofluorescence, which is a prerequisite for their interaction (Fig. 3E). Immunoprecipitation was the first classical experiment to study protein-protein interactions. We thus explored the interaction of β2-AR and PlexinA1 in MGC-803 cells through Co-IP and found that PlexinA1 and β2-AR bound with each other (Fig. 3G). These results suggested that PlexinA1 was one of the action targets of β2-AR. We also studied the β2-AR-PlexinA1 interaction in SGC-7901 cells and reached a consistent conclusion (Fig. 3A-D, Fig. 3G).

### PlexinA1 knockdown abolished β2-AR-mediated EMT activation in gastric cells treated with ISO

**Figure 4:**
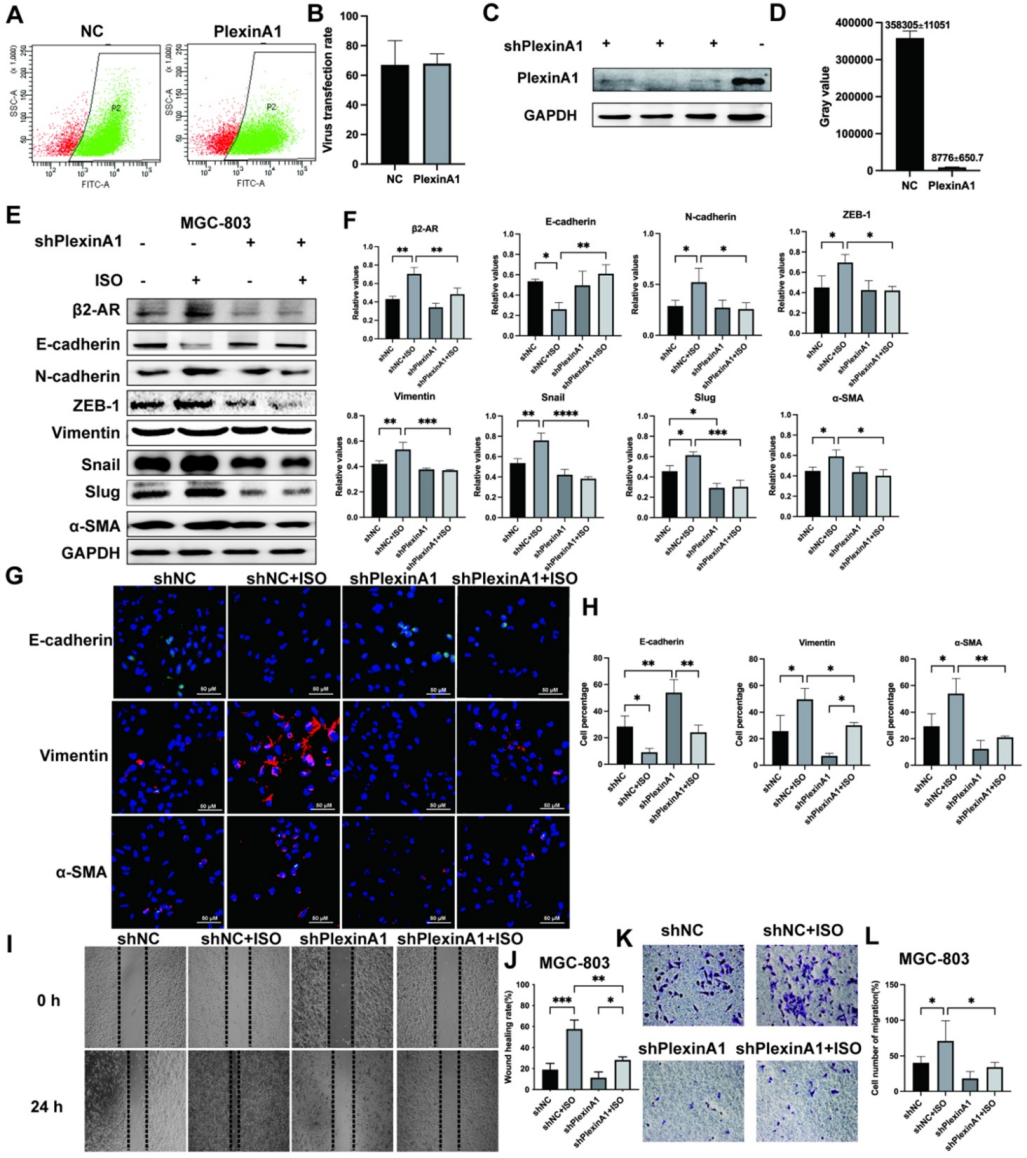
** Effects of interference with PlexinA1 through small hairpin RNAs (shRNAs) on the expression of PlexinA1.** The lentivirus infection rate confirmed by flow cytometry and statistical chart are shown in **(A-B)**. The protein bands and corresponding data analysis are shown in **(C-D)**. Effects of PlexinA1 interference through small hairpin RNAs (shRNAs) on the expression of β2-AR, an epithelial marker (E-cadherin) and mesenchymal markers (N-cadherin, ZEB-1, Vimentin, Snail, Slug and α-SMA). MGC-803 cells were treated with isoproterenol (ISO). The results for β2-AR, E-cadherin, N-cadherin, ZEB-1, vimentin, Snail, Slug and α-SMA were obtained by western blotting. Three independent repeated experiments were conducted. The gray values of the protein bands were analyzed through ImageJ software, relative values to glyceraldehyde-3-phosphate dehydrogenase (GAPDH) were calculated. The protein bands and associated statistical analysis data are shown in **(E-F)**. Furthermore, the expression of E-cadherin, Vimentin and α-SMA in different groups treated with PlexinA1 shRNAs was further confirmed by immunofluorescence and is shown in **(G-H)**. Finally, the scratch test and Transwell experiment examined the migration ability of MGC-803 cells. Typical images were shown in **(I-L)**. For the Transwell test, five fields were randomly selected under a light microscope, and the number of cells was counted. The data are presented as the mean±s.d. *, P < 0.05; **, P < 0.01; ***, P < 0.001; ****, P < 0.0001.

**Figure 5:**
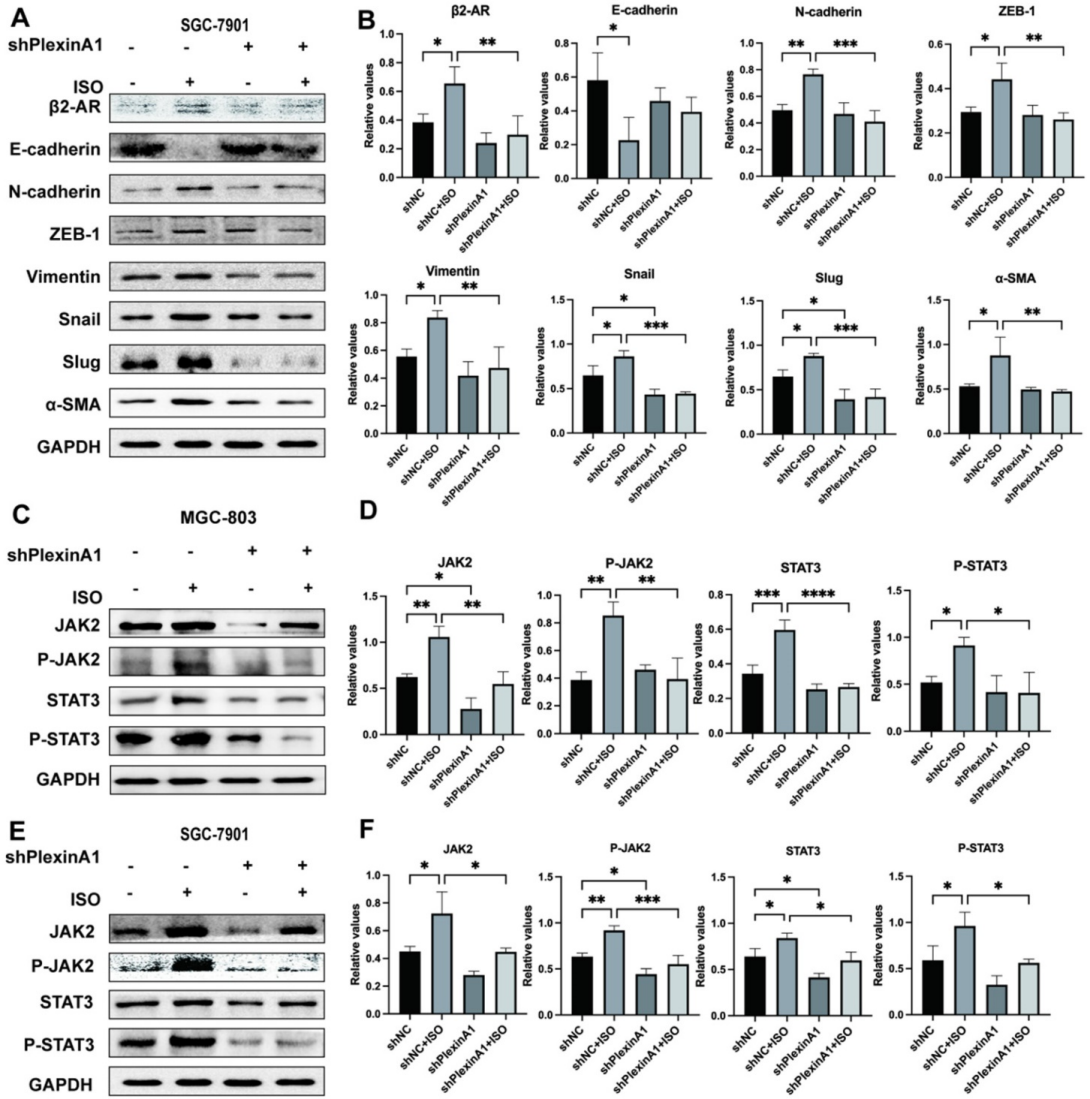
** The influence of interference with PlexinA1 through small hairpin RNAs (shRNAs) on the expression of β2-AR, epithelial markers (E-cadherin) and mesenchymal markers (N-cadherin, ZEB-1, Vimentin, Snail, Slug and α-SMA). SGC-7901 cells were treated with isoproterenol (ISO).** The results were obtained by western blotting. Three independent repeated experiments were conducted. The gray values of the protein bands were analyzed through ImageJ software, relative values to glyceraldehyde-3-phosphate dehydrogenase (GAPDH) were calculated. The protein bands and associated statistical analysis data are shown in **(A-B)**. The influence of PlexinA1 knockdown through small hairpin RNAs (shRNAs) on the protein levels of JAK2, P-JAK2, STAT3, and P-STAT3. The results of this study are shown in **(C-F)**. Three independent repeated experiments were conducted. The gray values of the protein bands were analyzed using ImageJ software, the values relative to glyceraldehyde-3-phosphate dehydrogenase (GAPDH) were computed. The data are presented as the mean±s.d. *, P < 0.05; **, P < 0.01; ***, P < 0.001; ****, P < 0.0001.

To further verify the role of PlexinA1 in β2-AR-mediated EMT, we knocked down the expression of PlexinA1 through small hairpin RNAs (shRNAs) in MGC-803 cells. The lentivirus infection rate reached 70%, as confirmed by flow cytometry, and the expression of PlexinA1 in MGC 803 cells treated with shRNAs is about 41 times less than that in MGC 803 cells treated with the negative control (Fig. 4A and D). The activation of β2-AR induced by ISO promoted EMT in MGC-803 cells. The knockdown of PlexinA1 blocked β2-AR-mediated EMT in MGC-803 cells, mainly manifested as increased epithelial markers such as E-cadherin and reduced mesenchymal markers including N-cadherin, ZEB-1, Vimentin, Snail, Slug and α-SMA (Fig. 4E and F). The expression of E-cadherin, Vimentin and α-SMA in different groups treated with or without PlexinA1 shRNAs was further confirmed by immunofluorescence (Fig. 4G and H). Moreover, the decrease in PlexinA1 via shRNAs could slow down the migration speed of MGC-803 cells induced by ISO as determined using a scratch test and Transwell experiment (Fig. 4I-L). We achieved the same results in SGC-7901 cells (Fig. 5A and B). Through these results, we demonstrated that PlexinA1 is closely involved in the regulation of EMT in both MGC-803 cells and SGC-7901 cells.

### PlexinA1 knockdown prevents the activation of JAK-STAT3 signaling induced by ISO

As a tyrosine kinase, JAK activates STAT3 and regulates gene expression, which plays important roles in various types of cancers 9, 10, 19-21. Our previous study found that β2-AR could activate STAT3 and initiate EMT in gastric cancer cells 4. However, the effects of β2-AR and PlexinA1 on JAK-STAT3 signaling remain to be further explored. In this study, our results showed that the activation of β2-AR induced by ISO increased the expression of JAK2, p-JAK2 (Tyr1007 and 1008), STAT3 and p-STAT3 (Tyr705) in MGC 803 cells (Fig. 5C and D), indicating that β2-AR plays important roles in the regulation of JAK-STAT3 signaling. Meanwhile, when the expression of PlexinA1 was inhibited by shRNAs, the phosphorylation activation of JAK-STAT3 signaling induced by ISO was blocked (Fig. 5C and D). Then, we obtained a consistent result in SGC 7901 cells (Fig. 5E and F). The results suggested that β2-AR exerts physiological functions by activating JAK-STAT3 signaling, which could be achieved through PlexinA1.

### JAK-STAT3 signaling mediates the occurrence of EMT in gastric cancer cells

**Figure 6:**
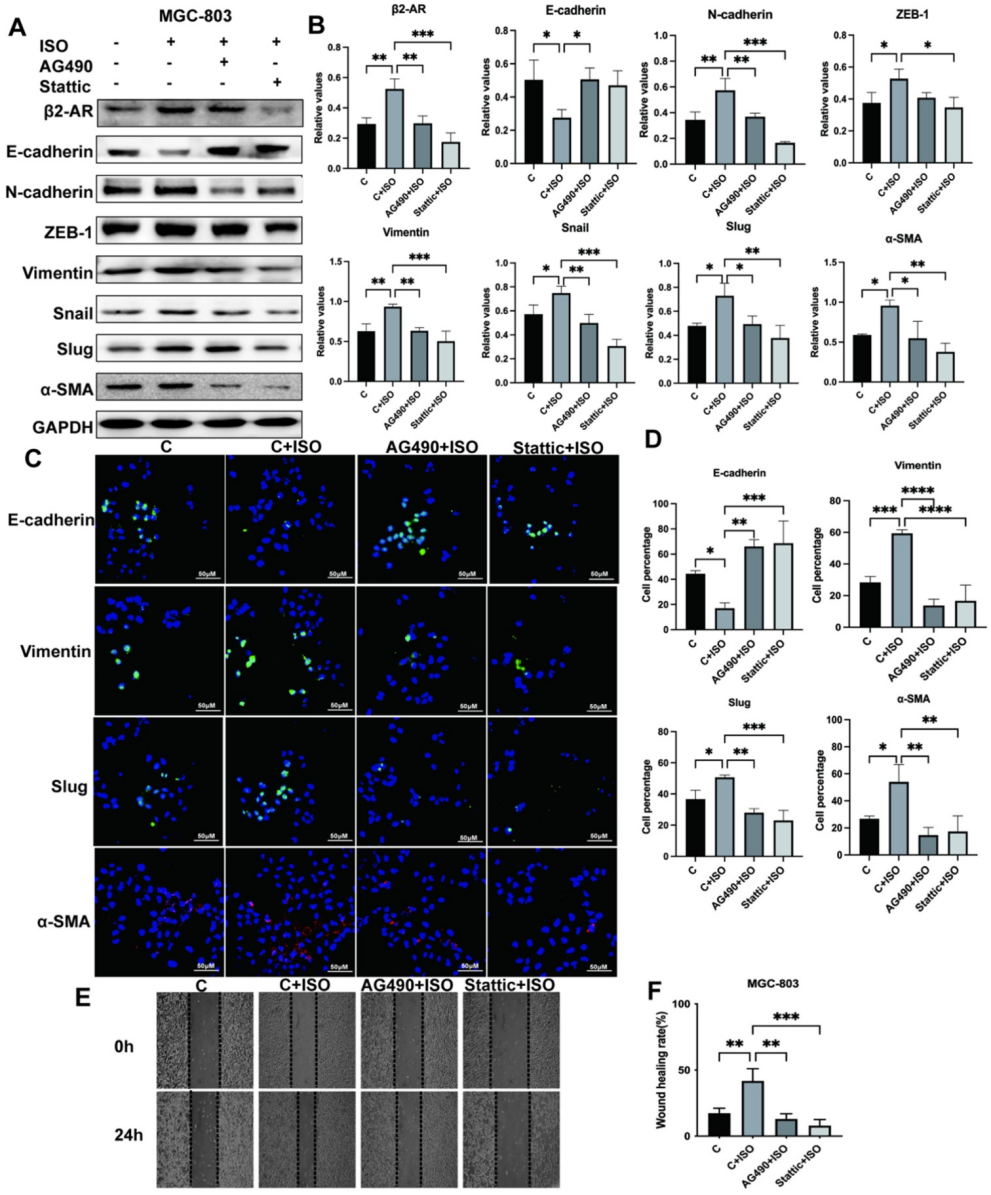
** Isoproterenol (ISO)-induced gastric cancer cell EMT was blocked via the JAK-STAT3 inhibitors AG490 and Stattic.** MGC-803 cells were treated with 20 µM isoproterenol (ISO), 20 µM AG490 or 20 µM Stattic for 12 hours, and the cells were collected for the next assay. The protein results for β2-AR, E-cadherin, N-cadherin, ZEB-1, Vimentin, Snail, Slug and α-SMA were obtained by western blotting. Three independent repeated experiments were conducted. The gray values of the protein bands were analyzed through ImageJ software, relative values to glyceraldehyde-3-phosphate dehydrogenase (GAPDH) were calculated. The protein bands and correlative statistical analysis data are shown in **(A-B)**. Additionally, the expression of E-cadherin, Vimentin, Slug and α-SMA in MGC-803 cells treated with ISO, AG490 and Stattic was confirmed by immunofluorescence, and obvious images are shown in **(C-D)**. Finally, scratch experiments were performed to investigate the migration ability of MGC-803 cells. Representative images are shown in **(E-F)**. The data are presented as the mean±s.d. *, P < 0.05; **, P < 0.01; ***, P < 0.001; ****, P < 0.0001.

The EMT process plays a critical role during cancer development through multiple pathways. Previous results suggested that β2-AR could pass STAT3 to promote EMT in gastric cancer cells. The relationship between JAK-STAT3 and EMT has not been thoroughly explored. This study adopted the JAK2 inhibitor AG490 and the STAT3 inhibitor Stattic to inhibit this pathway. Our research proved that ISO induced EMT of MGC-803 cells, while AG490 and Stattic reversed this phenomenon and inhibited the occurrence of EMT, as revealed by an increase in epithelial markers such as E-cadherin and a decrease in mesenchymal markers, including N-cadherin, ZEB-1, Vimentin, Snail, Slug and α-SMA, after blocking the JAK-STAT3 signaling pathway (Fig. 6A and B). Meanwhile, we used immunofluorescence to demonstrate the effect of the JAK-STAT3 inhibitor on EMT expression in MGC-803 cells and obtained consistent western blotting results (Fig. 6C and D). In addition, the results of scratch tests showed that JAK-STAT3 inhibitors were able to reduce the migration rate of MGC-803 cells promoted by ISO (Fig. 6E and F). Furthermore, we performed verification in SGC-7901 cells and obtained similar results (Fig. S2A-D). Our results indicated that the JAK/STAT3 signaling pathway was involved in the EMT induction process of gastric cancer.

## Discussion

The influence of psychosocial factors on the prognosis of cancer patients has received increasing attention. Studies have shown that chronic stress caused by long-term psychological stress, such as anxiety and depression, could promote the malignant progression of tumors by acting on β2-AR 8, 13, 14. However, the mechanism by which chronic psychological stress promotes the malignant progression of tumors is still unclear. At present, the clinic only provides general psychological support, such as health education and education for patients, and relatively precise intervention cannot be given. The influence of psychosocial factors on the prognosis of cancer patients has received attention. Studies have shown that chronic stress caused by long-term psychological stress, such as anxiety and depression, can promote the malignant progression of tumors by acting on β2-AR 22-25. It has been reported that in experimental models, blocking adrenergic receptors reverses the cancer-promoting effects of chronic stress 26. Furthermore, the downstream targets of β2-AR need further research. As suggested by these findings, ISO treatment did not significantly affect the ability of proliferation of gastric cancer cells. However, an increasing number of studies have revealed the important role of EMT in the invasion and migration ability of cancer cells 15-18. In this study, we confirmed that mimics of chronic stress affected EMT in gastric cancer cells.

Chronic stress affects the EMT of gastric cancer cells through multiple signaling pathways. Our previous studies have found that the β2-AR agonist isoproterenol affects the EMT of gastric cancer cells through STAT3-CD44. Other researchers have also found that ISO affects the EMT of gastric cancer cells through the β2-AR-HIF-1α-Snail signaling pathway, which in turn influences the invasion and migration ability of gastric cancer, and β2-AR can promote EMT by activating many pathways 3, 4, 18. The typical features of EMT are activation of ZEB1, N-cadherin, vimentin, Snail, Slug, and α-SMA and downregulation of the expression of the epithelial marker E-cadherin 15, 27. Our examination of the adopted β-adrenergic receptor agonist isoproterenol simulated chronic stress, which could promote EMT, and the inhibitor ICI 118,551 inhibited EMT in gastric cancer cells. Combining current research hotspots, focusing on chronic stress stimulating the secretion of adrenal hormones and acting on corresponding receptors, and further exploring its mechanism to promote the malignant progression of tumors constitute the research concept of “producing scientific hypotheses based on clinical issues”. PlexinA1 is a semaphorin family receptor that acts as an axon guidance factor. In recent years, PlexinA1 was found to play a critical role in regulating tumor cell migration and proliferation. A previous study suggested that the PlexinA1 expression level could be considered a potential diagnostic and prognostic marker in glioblastoma 28. A model for the establishment of immune-related genes for the prognosis of liver cancer found that PlexinA1 was considered an oncogene 29. We have reported the expression and clinical significance of gastric cancer 30. However, little is known about the relationship between PlexinA1 and tumor cell EMT. Preliminary work suggested that the β2-AR agonist isoproterenol could significantly promote the expression of PlexinA1 in gastric cancer cells and that PlexinA1 participates in the malignant progression of gastric cancer by mediating EMT and tumor angiogenesis in gastric cancer cells. It is relevant in regulating the malignant progression of gastric cancer 8, 18. However, the interaction between PlexinA1 and β2-AR has not been revealed. Our research results showed that isoproterenol could significantly promote the expression of PlexinA1 in gastric cancer cells and promote EMT in gastric cancer cells. It has also been newly discovered that PlexinA1 and β2-AR are colocalized and colocalized in space. By examining interactions, this study showed that PlexinA1 might be a potential target for β2-AR to regulate the malignant progression of gastric cancer.

**Figure 7:**
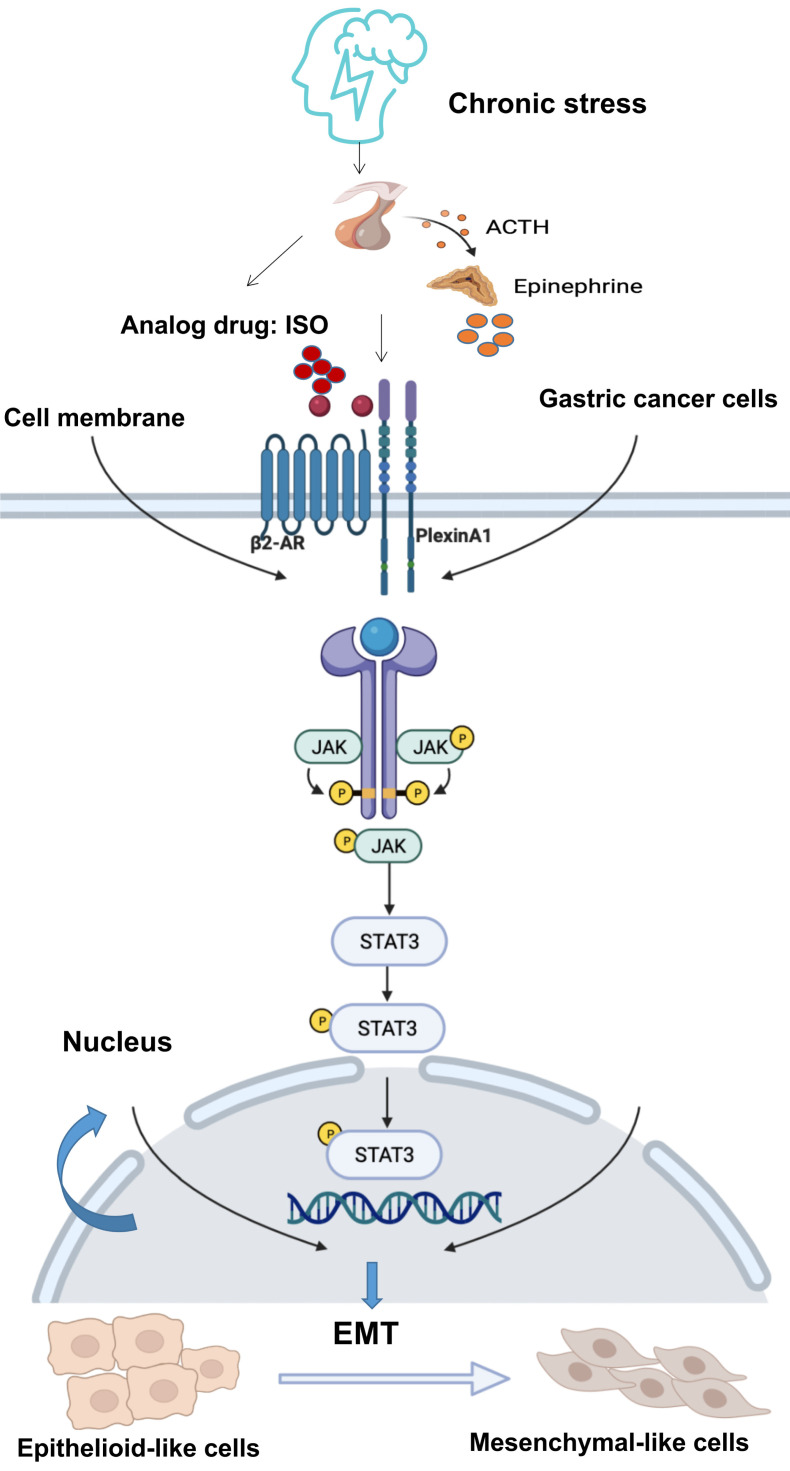
** Schematic diagram of the β2-AR-PlexinA1-JAK/STAT3 pathway.** The schematic diagram summarizes the results of this study. Our research showed that chronic stress-related hormones are related to the occurrence of EMT in gastric cancer. According to our observations, chronic stress then triggers the HPA (hypothalamus-pituitary-adrenal) axis to promote the release of catecholamine hormones. These β2-AR agonists not only recognize β2-AR but also activate the transmembrane protein PlexinA1. β2-AR interacts with PlexinA1 and subsequently promotes the downstream JAK-STAT3 signaling pathway. The JAK-STAT3 signaling pathway mediates the phenotypic transition of gastric cancer cells to EMT and subsequently stimulates the undesirable progression of human gastric cancer.

The JAK/STAT3 signaling pathway plays a vital role in various types of cancer. Activation of the JAK/STAT3 signaling pathway is related to EMT, tumor invasion and metastasis 9, 19-21, 31. It has demonstrated that β2-AR shows kinase activity 25, 26. β2-AR could induce the threonine phosphorylation of the IL-2Rβ, and further activated JAK-STAT3 32. Besides, in tumor cells, chronic stress mediated by β2-AR promoted the release of IL-6 and activated the JAK-STAT3 signaling pathway 33. There is no evidence that PlexinA1 has kinase activity. Through our study, PlexinA1 regulated the JAK-STAT3 signaling pathway through the interaction with β2-AR kinase. In this study, when EMT induced by PlexinA1/β2-AR promoted the migration of gastric cancer cells, the JAK/STAT3 pathway was activated, and AG490 and Stattic reversed these effects. Overall, the results showed that EMT induced by PlexinA1/β2-AR was inhibited, thus confirming that the JAK/STAT3 pathway is involved in EMT induction. Overall, our study solved the molecular mechanism of chronic stress-mediated EMT through PlexinA1 at the cellular level and further explored the role of β2-AR and PlexinA1 in chronic stress-promoted malignant progression of gastric cancer. The interaction of β2-AR and PlexinA1 activated the JAK-STAT3 signaling pathway and promoted EMT in gastric cancer cells. (Fig. 7) According to our observations, interference with the key target of the signaling pathway, PlexinA1, provides new treatments for gastric cancer patients with clinically unhealthy emotions.

Our study explored whether chronic stress promotes epithelial-mesenchymal transition through the PlexinA1/β2-AR-JAK-STAT3 signaling pathway in two types of gastric cancer cells, which was not verified *in vivo* and requires further research. According to our observations, PlexinA1 and β2-AR interact; however, it is unknown how the two regulate downstream pathways, which can be further considered.

## Conclusion

Taken together, chronic stress induces epithelial-mesenchymal transition through the PlexinA1/β2-AR-JAK-STAT3 signaling pathway in gastric cancer cells. Isoprenaline, a β2-AR agonist, recognizes and activates β2-AR/PlexinA1, which subsequently activates the downstream JAK-STAT3 signaling pathway. This series of activations functions as a positive regulator of EMT under chronic stress. Our findings revealed the key role of PlexinA1 in chronic stress-induced EMT. The results provide a new treatment for gastric cancer patients with chronic stress by intervening in the key target of the signaling pathway, PlexinA1.

## Supplementary Material

Supplementary figures.
