# Supplementary material for: PlexinA1 activation induced by β2-AR promotes epithelial-mesenchymal transition through JAK-STAT3 signaling in human gastric cancer cells
Source: J Cancer. 2022 Apr 18;13(7):2258–70. doi: 10.7150/jca.70000 (PMC9066200; doi:10.7150/jca.70000)
Supplement: Supplementary file 1 — Supplementary figures. [file jcav13p2258s1.pdf]

Figure S1. Effects of isoproterenol (ISO) and ICI118,551 (ICI) on the expression of β2-AR, an epithelial marker (E-cadherin) and mesenchymal markers (N-cadherin, ZEB-1, Vimentin, Snail, Slug and α-SMA). SGC-7901 cells were treated with 0, 2μM, 10μM, or 20μM ISO or 20μM, 40μM, or 50μM ICI, and the cells were collected for detection 12 or 24 hours later. The protein levels of β2-AR, E-cadherin, N-cadherin, ZEB-1, Vimentin, Snail, Slug and α-SMA were determined by western blotting. Three independent repeated tests were conducted. The gray values of the protein bands were analyzed through ImageJ software, relative values to glyceraldehyde-3-phosphate dehydrogenase (GAPDH) were calculated. The protein bands and associated statistical analysis data in the ISO group are shown in (A-B), and those in the ICI group are presented in (C-D). The data are presented as the mean±s.d. *, $P < 0.05$; **, $P < 0.01$; ***, $P < 0.001$; ****, $P < 0.0001$.

Figure S2. Isoproterenol (ISO)-promoted gastric cancer cell EMT was ameliorated by the JAK-STAT3 inhibitors AG490 and Stattic. SGC-7901 cells were treated with 20μM isoproterenol (ISO), 20μM AG490 or 20μM Stattic for 12 or 24 hours, and the cells were collected for the next assay. The protein results for β2-AR, E-cadherin, N-cadherin, ZEB-1, Vimentin, Snail, Slug and α-SMA were obtained by western blotting. Three independent repeated experiments were conducted. The gray values of the protein bands were analyzed through ImageJ software, relative values to glyceraldehyde-3-phosphate dehydrogenase (GAPDH) were calculated. The protein bands and correlative statistical analysis data are shown in (A-B). Then, scratch tests were performed to determine the migration ability of SGC-7901 cells. Images are shown in (C-D). The data are presented as the mean±s.d. *, $P < 0.05$; **, $P < 0.01$; ***, $P < 0.001$.

Figure S1

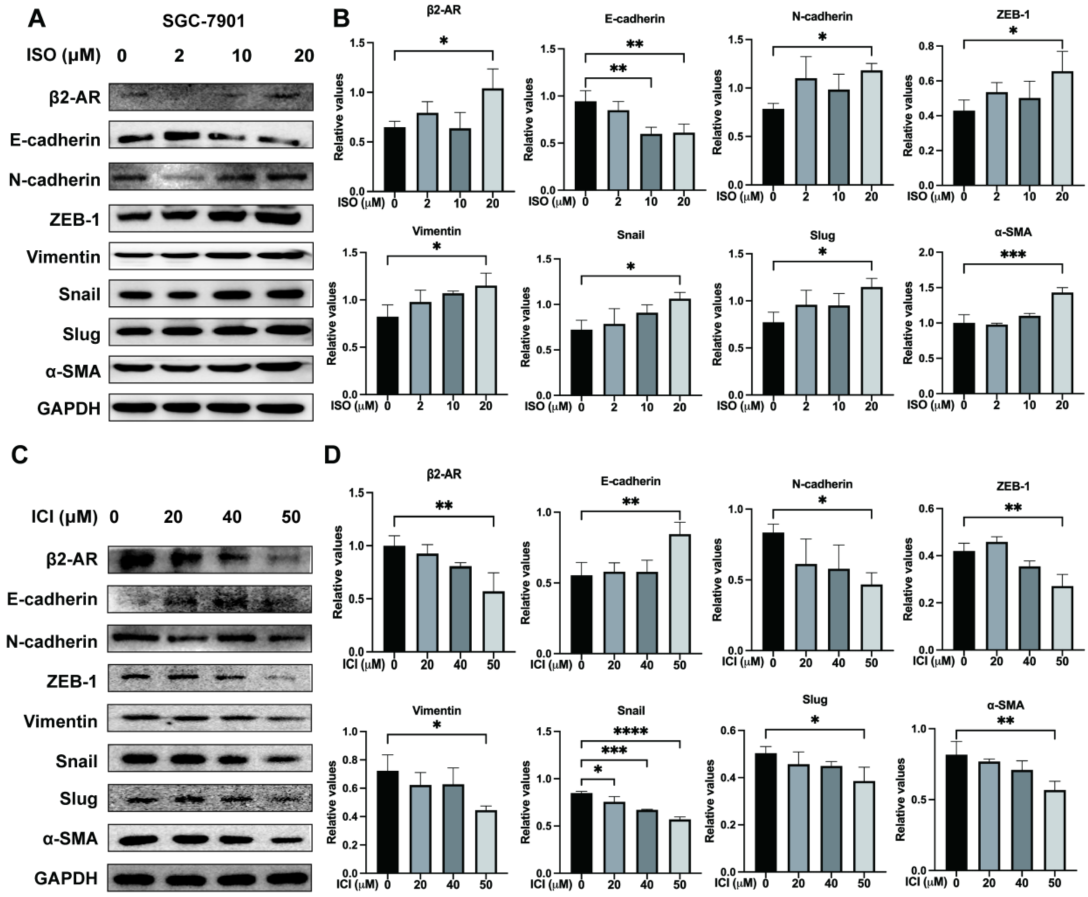

A
SGC-7901
ISO (μM) 0 2 10 20
β2-AR
E-cadherin
N-cadherin
ZEB-1
Vimentin
Snail
Slug
α-SMA
GAPDH
B
β2-AR
E-cadherin
N-cadherin
ZEB-1
Vimentin
Snail
Slug
α-SMA
Relative values
ISO (μM) 0 2 10 20
C
ICI (μM) 0 20 40 50
β2-AR
E-cadherin
N-cadherin
ZEB-1
Vimentin
Snail
Slug
α-SMA
GAPDH
D
β2-AR
E-cadherin
N-cadherin
ZEB-1
Vimentin
Snail
Slug
α-SMA
Relative values
ICI (μM) 0 20 40 50

Figure S2

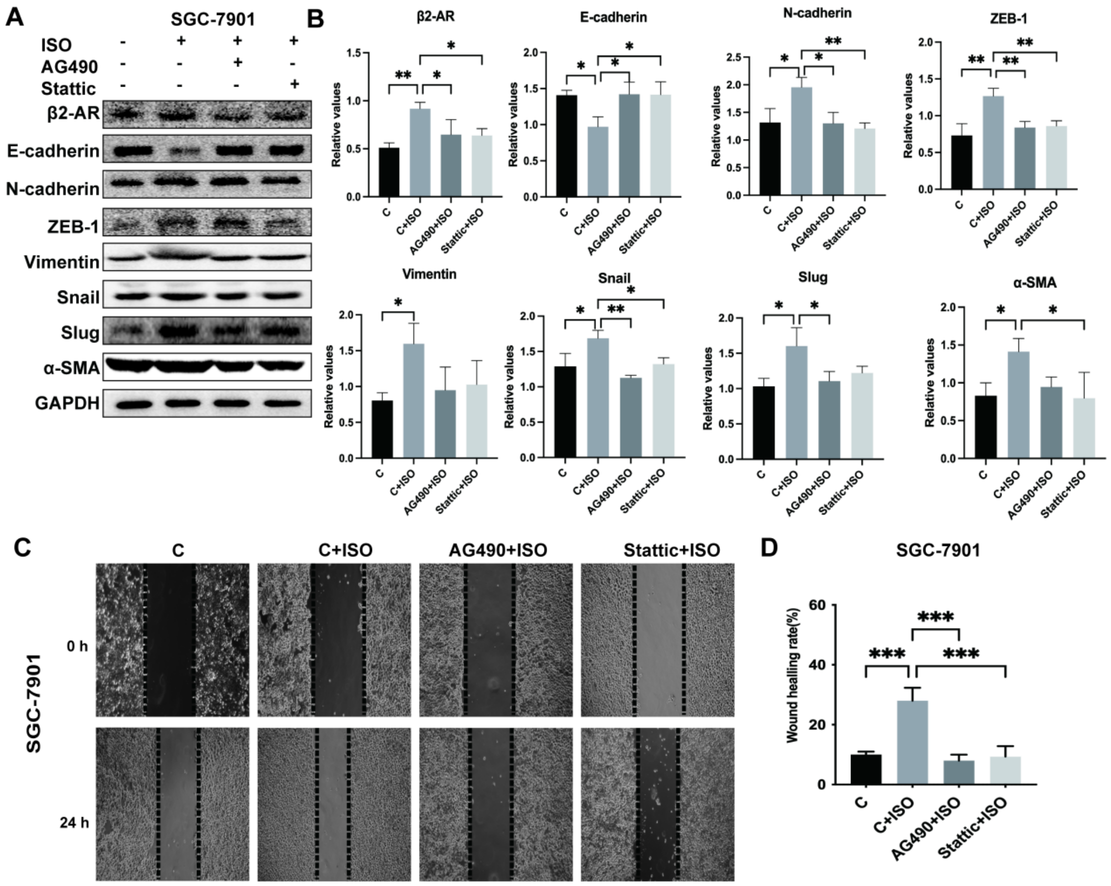
A
SGC-7901
ISO
AG490
Stattic
β2-AR
E-cadherin
N-cadherin
ZEB-1
Vimentin
Snail
Slug
α-SMA
GAPDH
B
β2-AR
E-cadherin
N-cadherin
ZEB-1
Vimentin
Snail
Slug
α-SMA
Relative values
C
C+ISO
AG490+ISO
Stattic+ISO
C
0 h
24 h
SGC-7901
D
SGC-7901
Wound healling rate(%)
